# An Investigation into the Surface Integrity of Micro-Machined High-Speed Steel and Tungsten Carbide Cutting Tools

**DOI:** 10.3390/mi14101970

**Published:** 2023-10-22

**Authors:** Minh Nhat Dang, Surinder Singh, John H. Navarro-Devia, Hannah J. King, Rosalie K. Hocking, Scott A. Wade, Guy Stephens, Angelo Papageorgiou, James Wang

**Affiliations:** 1 The Australian Research Council (ARC) Training Centre in Surface Engineering for Advanced Materials (SEAM), School of Engineering, Swinburne University of Technology, P.O. Box 218, Hawthorn, VIC 3122, Australia; 2 Sutton Tools, 378 Settlement Rd, Thomastown, VIC 3074, Australia

**Keywords:** micro machining, cutting tools, tungsten carbide, high-speed steels, surface roughness

## Abstract

The performance and lifespan of cutting tools are significantly influenced by their surface quality. The present report highlights recent advances in enhancing the surface characteristics of tungsten carbide and high-speed steel cutting tools using a novel micro-machining technique for polishing and edge-honing. Notably, the main aim was to reduce the surface roughness while maintaining the hardness of the materials at an optimal level. By conducting a thorough analysis of surfaces obtained using different techniques, it was found that the micro-machining method effectively decreased the surface roughness of the cutting tools the most effectively out of the techniques investigated. Significantly, the surface roughness was reduced from an initial measurement of 400 nm to an impressive value of 60 nm. No significant change in hardness was observed, which guarantees the maintenance of the mechanical properties of the cutting tools. This analysis enhances the comprehension of surface enhancement methodologies for cutting tools through the presentation of these findings. The observed decrease in surface roughness, along with the consistent hardness, exhibits potential for improving tool performance. These enhancements possess the capacity to optimise manufacturing processes, increase tool reliability, and minimise waste generation.

## 1. Introduction

Cutting tools have played a crucial role in numerous manufacturing sectors, facilitating the manipulation, processing, and refinement of a wide range of materials. The composition and technological methods utilised in the manufacturing and finishing processes of these tools have a significant impact on their quality, efficiency, and durability. The materials of high-speed steels (HSS) and tungsten carbides (WC) have garnered significant attention in this field due to their notable contributions to the cutting tool industry. The alloy known as HSS consists of tungsten, chromium, molybdenum, vanadium, and occasionally cobalt. It exhibits exceptional wear resistance and retains its hardness even when exposed to high temperatures, rendering it suitable for a wide array of applications [1]. Tungsten carbides, when incorporated into composite materials alongside cobalt or nickel as a binding agent, exhibit remarkable hardness and resistance to abrasion [2]. As a result, they have become indispensable in the realm of machining, particularly for tasks that require high levels of performance and durability [3,4,5].

To meet the expectations of the most demanding customers, cutting tools must possess optimal mechanical properties, such as high toughness, hot hardness, and wear resistance. One of the ways to enhance these mechanical properties of the tools and improve the surface quality of the machined components is to carefully control the surface topography and nose radius of the cutting edge of the tool, and then application of suitable hard coatings, which improves the tool’s workability [6]. For instance, HSS and WC cutting tools can be coated with different nitrides, such as titanium nitride and its derivatives, to increase wear resistance and anti-corrosion ability, enhancing the tribological compatibility within the cutting tool/workpiece system [7,8]. To maximize the benefits of coatings, the tool surface must undergo pre-treatment through surface finishing techniques, which can eliminate defects, reduce roughness, and enhance the tool’s quality and performance [9].

Similar to its application in precision additive manufacturing, micro-machining has become a crucial technique in the field of surface finishing, as it provides a means to achieve the precise manipulation of surface topography and facilitates the elimination of material at a micro or nano-scale [10,11]. Methods such as micro-grinding, micro-milling, and micro-polishing are employed to modify surface structure of the tools to achieve a well-defined cutting edge, and reduce any defects that can significantly affect performance [12,13]. Within the cutting tool industry, a variety of surface treatment methods with suitable machining parameters have been developed and implemented over an extended period of time [14,15,16,17]. The aforementioned processes can be categorised into three distinct approaches: mechanical, chemical, and electrochemical. Mechanical techniques, including grinding, honing, and lapping, have been widely employed due to their ability to effectively eliminate substantial amounts of material, diminish surface roughness, and establish distinct surface characteristics. Chemical methodologies such as etching, pickling, and passivation have demonstrated enhanced efficacy in the elimination of surface impurities and the enhancement of surface quality. Electrochemical techniques, such as electro-polishing and electrochemical grinding, are commonly used due to their inherent attributes of precision and accuracy in generating a refined and uniform surface texture. Aside from that, an ultrashort laser-induced source can be implemented to tailor the surface properties, resulting in the significant impact on the microtextures on these ultrahard materials [18].

The evaluation of the efficacy of a specific surface finishing procedure can be conducted in terms of surface roughness. Surface roughness is a term used to describe the deviations from a smooth surface that occur as a result of the machining process [19,20]. These abnormalities can have a notable impact on the performance of the tool being used. The average roughness (Ra) is the predominant measure employed for assessing surface roughness. It is determined by calculating the arithmetic mean of peak height (Rp) and valley depth (Rv). The term “Rq” denotes the root-mean-square roughness of the Rp and Rv values. Rt is defined as the overall height of the roughness profile, encompassing the disparity between the height of the highest peak Rp and the depth of the deepest valley within the specified evaluation length. On the other hand, Rz represents the maximum height of the roughness profile, calculated by summing the absolute values of the heights of the highest-profile peaks and the depths of the deepest valleys within the evaluation length. Herein, it is imperative to ascertain the suitable surface finishing methodology that would provide the intended surface roughness and augment the efficacy of the tool. The significance of this matter is particularly pronounced in the contemporary competitive landscape of the sector, wherein tool producers make considerable efforts to provide the highest-quality products that meet customers’ preferences and requirements. Numerous scholarly investigations have examined diverse methodologies pertaining to the process of surface finishing. For instance, Fang et al. reported that laser finishing on carbide materials led to an increase in surface roughness by roughly an order of magnitude compared to mirror-polished surfaces [21]. The observed rise in magnitude can be ascribed to the creation of a molten layer followed by its subsequent redeposition. Additionally, they found a significant enhancement in hardness following laser treatment. In another example, Tillmann and colleagues made the observation that heat-treated HSS has the potential to attain a surface finish similar to that achieved by metallographic polishing methods when employed in meso- and micromilling setups [22]. It is worth mentioning that mesomilling demonstrated the presence of cutting grooves, whereas micromilling resulted in a topography that was devoid of defects, exhibiting an average roughness measuring below 15 nm. The micromilling process resulted in the generation of significant compressive residual stresses (σ ~ −800 MPa) which exhibited a quasi-isotropic nature. The team led by Riu et al. utilized the technique of dry electropolishing to attain surfaces that are resistant to corrosion, exhibiting a roughness level measuring less than 9 nm [23]. Furthermore, they were able to maintain consistent values for hardness and elastic modulus throughout the process. The aforementioned findings on the plain surface indicate a range of potential pathways that show promise in achieving high-quality surface finishes across the complex geometry of the same hard tool materials.

Through the implementation of appropriate surface finishing processes, manufacturers have the ability to enhance the quality and visual appeal of their cutting tools. This, in turn, increases productivity, decreases energy consumption, and produces high-quality products. Also, the substrate’s final topography and residual stresses are affected by the edge preparation performed prior to the coating process. Numerous studies have demonstrated the benefits of edge preparation of cutting tools regardless of their cutting operations [24,25]. Based on these findings, it is clear that proper preparation of the cutting edge will improve cutting performance by increasing the mechanical strength and stability of the cutting edge. Pre-treatment improves the tool’s wear resistance by enhancing its mechanical strength and decreasing the likelihood of chipping and first crack formation along the edge. In addition, cutting edge preparation affects adhesion strength; therefore, a smooth cutting-edge surface facilitates coating application. The ideal cutting edge form for a given machining process depends on the machining conditions as well as the characteristics of the tool and the material being machined or precisely micro-machined [26]. One example of a study conducted by Li et al. has shown that the utilization of chamfered inserts has a tendency to yield a more refined surface finish on the machined component, along with a notable decrease in surface roughness [27]. In addition to this, Uhlmann et al. discovered that enhancements in surface roughness are not solely associated with less tool wear but are also impacted by the cutting edge radius [28]. It is evident that the implementation of an optimum edge preparation has the potential to result in improved machining performance, encompassing the enhancement of the workpiece surface quality. Nevertheless, it is important to acknowledge that whereas edge preparation procedures can really enhance surface roughness to a considerable extent, they seldom attain ultra-low Ra values at the nanoscale. Typically, these approaches yield surface roughness at the sub-micron level. This constraint implies that although edge preparation has the potential to enhance performance, its ability to enhance surface finish may be limited by the inherent constraints of mechanical surface finishing methods.

Therefore, in this study, we investigate the surface morphological, elemental, and mechanical properties of raw and micro-machined HSS and WC tools that have obtained a nano-scale roughness level. By examining the micro-machined tool surface integrity, we aim to gain a better understanding of the polishing mechanism and how it might assist the improvement in tool performance.

## 2. Materials and Methods

The cutting tools employed in this investigation comprised two distinct materials: M2 HSS and WC (8% Co), both of which were provided by Sutton Tools (Melbourne, Australia). These tools’ surfaces were refined employing a proprietary micro machining process, the details of which have been kept confidential due to industry partner restrictions. Before conducting additional micro-structure analysis, the tool samples were cross-sectioned using a Struers (Milton, Queensland, Australia)’s B0D20 diamond-embedded cutting wheel on a Setocom-50 cutter machine operating at the default direct-cut speed (2200 rpm, 0.060 mm/s). The cut samples were subsequently mounted using a mixture of epoxy resin, which was allowed to cure for a period of 48 h. The samples that were affixed to a mount were subsequently subjected to grinding using silicon carbide grinding papers with grit sizes ranging from #220 to #1200. Additionally, diamond suspensions with particle sizes of 9 and 6 µm, provided by Struers, were utilized for the grinding process, with the specific choice of suspension dependent on the analysis technique employed.

Optical images were captured on the DSX1000 digital microscopes with 20× Olympus lens, provided by Evident (Sydney, Australia). The Zeiss Supra 40 VP FE-SEM was utilised for the Scanning Electron Microscopy (SEM) analysis, specifically for the characterisation of rod samples, in this study. The rods were being subjected to both longitudinal and transverse cutting techniques, and the resultant transparencies were being cleansed using deionized water and acetone. The transparencies that had been washed and sliced were subsequently attached to a copper substrate using carbon adhesive. The FE-SEM analysis of high-speed steel was performed using a beam of 5 kV, whereas the characterisation of tungsten carbide was carried out at 10 kV. The energy dispersive X-ray spectroscopy (EDX) technique was utilised to analyse both sample materials, employing a voltage of 20 kV and taking approximately 3 min per scan and 5 relocating scans per spot.

The Bruker D2 Phaser Powder X-ray Diffraction (XRD) system was utilized to perform XRD analysis. The experiment employed a cobalt (Co) X-ray source with a wavelength of 1.79026 Å, operating at a voltage of 30 kV and a current of 10 mA. The slit was set at a fixed angle of 1°. The system was outfitted with a LYNXEYE XY-T detector operating in 1D mode, in conjunction with a 2.5° Soller module. The scanning parameters were configured to initiate at an angle of 25° and terminate at 120°, utilising a step size of 0.02° and a dwell period of 1 s for each step. The rotational velocity was consistently held at 0 rpm. The data were acquired using the Bragg–Brentano geometry in a continuous mode, and afterwards processed with the DIFFRAC.EVA V5 software, then converted to equivalent patterns obtained by Cu source via PowDLL Converter V3.0 software to evaluate the crystalline structures and phase compositions.

A 3D profilometer is a surface measurement instrument that utilises non-contact methods to provide a highly accurate and precise representation of surface topography in three dimensions. To obtain accurate and precise measurements, the surface of the sample was initially subjected to a cleaning process in an ultrasonication bath, using a combination of distilled water and acetone. The cleansing procedure facilitates the elimination of contaminants and the establishment of a consistent surface for subsequent examination. The surface of the sample was subsequently analysed using a Bruker ContourGT-X 3D Optical Profiler in VSI mode, with a magnification of 50×. The roughness data were subsequently analysed using the Vision84 software with automatic Remove Tilt and Gaussian Regression Filters (short wavelength cut-off pass of 0.025 mm, tolerance 0.001%), which adheres to the ISO 4288 standard [29]. The purpose of this standard is to establish a set of guidelines for the measurement and assessment of surface roughness metrics. These guidelines aim to promote uniformity and precision in measurements conducted by different devices and operators. A set of ten random measurements were performed for each sample to obtain statistically significant results, with the roughness and standard deviation subsequently computed. The calculation of average roughness for certain samples can be conducted by performing a scan over an area of 0.5 × 0.5 mm^2^ in stitching mode. The utilisation of the stitching mode facilitates the achievement of a comprehensive and precise representation of surface intricacy.

The Duramin-40 Hardness Tester is a Vickers microhardness testing instrument. It is equipped with a diamond indenter that allows precise loads of 300 gf for both HSS and WC to be applied, dwell time of 10 s as well as a high-resolution camera that can capture images of the indents for subsequent analysis. The machine is also furnished with Duramin software that calculates hardness values automatically based on the size of the indentations.

## 3. Results and Discussion

**Figure 1:**
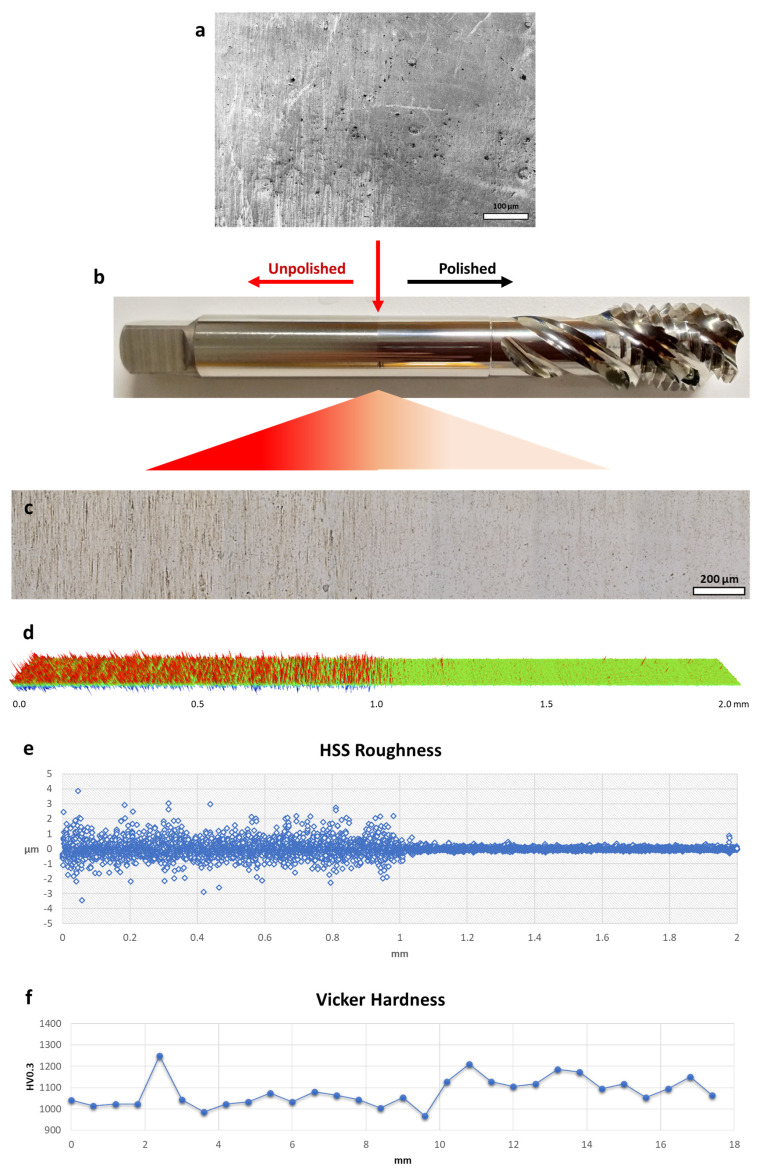
Polished–unpolished transition zone of HSS tool sample after micro-machining process: (**a**) SEM photo, (**b**) photograph of the drill bit investigated, (**c**) optical photo, (**d**) 3D profilometer photo with area of 0.12 × 2.0 mm^2^, (**e**) roughness change chart and (**f**) microhardness measurement on the same zone.

**Figure 2:**
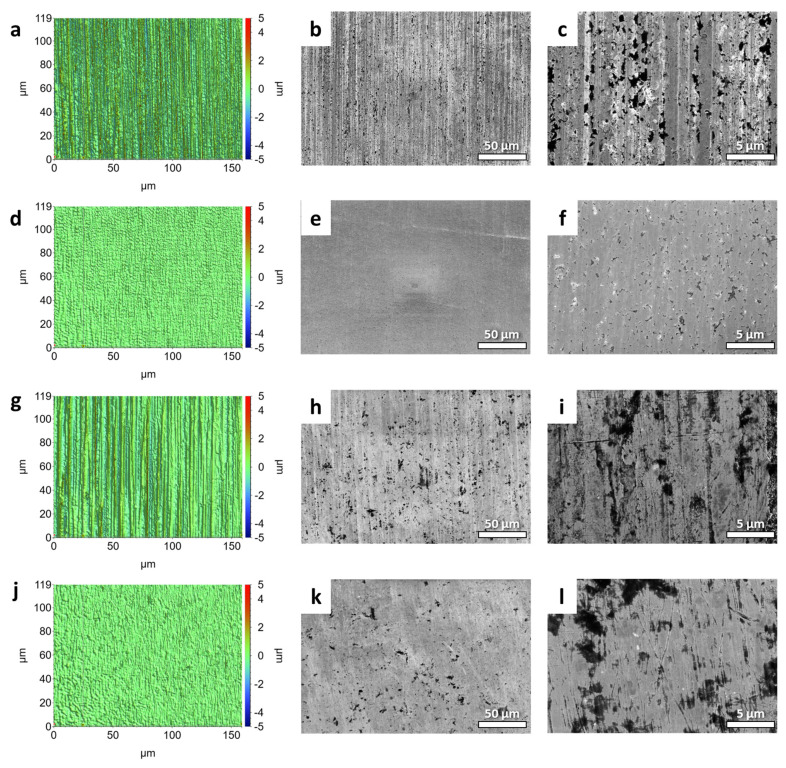
(**a**,**d**,**g**,**j**) 3D profilometer and (**b**,**c**,**e**,**f**,**h**,**i**,**k**,**l**) SEM images of (**a**–**c**) unpolished and (**d**–**f**) polished WC sample; (**g**–**i**) unpolished and (**j**–**l**) polished HSS sample.

Preliminary examinations were performed on the micro-machined HSS sample at the unpolished-polished transition zone (UPTZ) utilising different microscopy analyses. The unpolished section exhibited discernible grinding lines, which are commonly associated with CNC production methods. Additionally, the presence of holes and burrs was detected in the SEM and optical microscopy (Figure 1a–c). In sharp contrast, the defects were conspicuously lacking in the polished area. The visual evaluation readily discerns the heightened reflection and gloss of the polished segment in contrast to its unpolished counterpart. The results of quantitative assessments on surface roughness demonstrate a notable decrease following the micro-machining process. The utilisation of 3D profilometer investigations provided confirmation of the improved surface quality, thereby demonstrating an extraordinarily low roughness value that extends to the nanoscale (Figure 1d). Nevertheless, there were still detectable tiny surface defects on the polished surface. Following the process of micro-machining, the distribution of roughness values exhibited a greater degree of homogeneity, characterised by a mean value just over 74.3 nm and a standard deviation of 10.3 nm. In comparison, the pre-machining roughness exhibited a mean value of around 269.0 nm, accompanied by a standard deviation of 16.2 nm. Significantly, the Rz value exhibited a noticeable decrease from around 10 µm to a mere 2 µm, while consistently maintaining a stable standard deviation (Figure 2a,d). The grinding texture diminished noticeably, and concurrently, the hole size reduced across the entire polished region, as evident in the resulting photos (Figure 2b,c,e,f). These visual observations corroborate the roughness data presented in the graph. The data presented indicate that the micro-machining procedure successfully decreased the maximum heights while simultaneously increasing the depths on the surface of the tool (Figure 1e). It is noteworthy that the micro-hardness exhibited a very consistent trend, showing just a slight rise of around 11% following the micro-machining process (Figure 1f). The potential cause of this phenomenon might be ascribed to the removal of the grinding-burn layer, which is a common side-effect of the CNC procedure [30]. The micro-machining technique appeared to have removed the outer layer, which had a deceptively softer texture, revealing the essentially tougher structure of the underlying high-speed steel core. When it comes to producing high-quality finishes, our findings hold fairly well in comparison to those of other studies. At 370 V and 120 mS/cm of electrolyte conductivity, Schulze et al. were able to reduce the surface roughness of tool steel from 300 nm to 67 nm in 240 s using electrochemical pulse polishing [31]. Surface roughness as low as 0.5 µm was achieved by Meylan et al. using high-power diode laser polishing at a wavelength of 980 nm [32]. At a translating speed of less than 1 mm/s, Kawanaka and Kunieda were able to create a mirror-like surface smoother than Rz 0.2 mm using a monopolar pulse current with a short duration of 100 ms and a low duty factor under 10% [33]. Their breakthrough performance was traced back to the fact that they worked in an enclosed system with flat samples, which has advantages over the complex tool used in our study.

**Figure 3:**
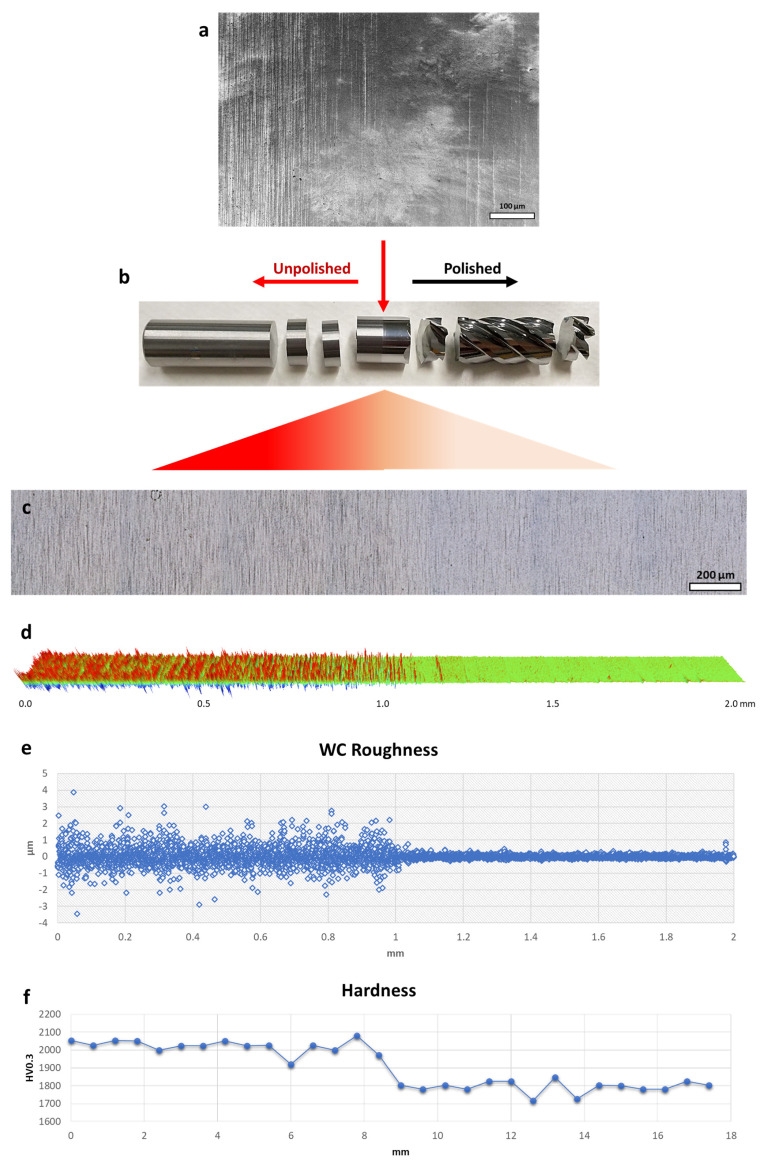
Polished—unpolished transition zone of WC tool sample after micro-machining process: (**a**) SEM photo, (**b**) photograph of the drill bit investigated, (**c**) optical photo, (**d**) 3D profilometer photo with area of 0.12 × 2.0 mm^2^, (**e**) roughness change chart and (**f**) microhardness measurement on the same zone.

Similar to the observations made on the high-speed steel, the tungsten carbide sample displays noticeable visual changes resulting from the micro-machining process (Figure 3b). These changes are evident in the form of a more pronounced and darker coloration in the polished area, highlighting the different reactions of various materials to comparable mechanical interventions. Significantly, although the grinding lines exhibit a noticeable reduction, they were not completely eradicated, and so emphasised the distinction between the prominent, more profound grinding textures and the more delicate, superficial textures: the former endured, while the latter were meticulously refined (Figure 3c). The intricate behaviour seen can be ascribed to the intricate microstructural composition of tungsten carbide. Carbides, renowned for their intrinsic ceramic nature, have a structure commonly composed of cemented binders, such as cobalt (Co), intricately dispersed amid tungsten (W) particles. The incorporation of a composite structure confers distinct mechanical and chemical properties to the material. In contrast to the generally uniform HSS matrix, the WC framework, either ultra-fine or extra-coarse type, poses difficulties when exposed to procedures that may result in cobalt leaching. During the process of leaching, the resulting voids remain unfilled and might expand due to the inherent nature of the ceramic matrix [34]. The process of micro-machining has been observed to worsen cobalt depletion, resulting in the displacement or migration of nearby tungsten particles as well as the creation of voids in the binder material, while simultaneously improving the surface texture. The observed morphological progression, when examined through a mechanical lens, leads to a measurable decrease of approximately 20% in the hardness of the polished region (Figure 3f). This reduction indicates the presence of inherent microscopic alterations caused by the process of micro-machining. From a topological perspective, it can be observed that post-machining valleys have increased depth, while peaks are reduced in magnitude. This is evidenced by a significant decrease in the Rz value from 8.5 µm to 1.5 µm, as well as a decrease in Ra from 396.0 nm to 63.7 nm (Figure 2g,j). The aforementioned changes were accompanied by a decrease in the standard deviation from 13.8 nm to 9.9 nm in average roughness, as clearly depicted in the roughness distribution chart of the UPTZ (Figure 3d,e). In comparison to previous investigations on the polishing of carbide materials, our micro-machining methodology presented a comparable degree of surface enhancement. An example of significant progress in surface roughness reduction was demonstrated by Kumar et al., who successfully achieved a 27% enhancement by employing abrasive flow polishing, resulting in the reduction in Ra to sub-micron levels [35]. In contrast, Lyu et al. utilized a brush tool-assisted shear-thickening polishing technique, which resulted in a remarkable Ra of less than 10 nm. It is important to note, however, that this achievement was limited to the uppermost surface region. This limitation arose from a decrease in slurry flow velocity caused by the presence of agglomerates, which in turn affected the cutting teeth of the insert. Consequently, the polishing process exhibited reduced efficiency in the region near the cutting edge of the root [36]. Furthermore, Lv et al. conducted a study in which they investigated different combinations of media for drag finishing on carbide tools. Their objective was to determine the optimal conditions that would result in minimal microchipping and a surface roughness of less than 0.1 µm [37]. Although each of these methods has its own unique advantages, our micro-machining process offers a well-rounded approach that effectively tackles both surface roughness and the reduction in microchipping. This makes it a versatile choice for complex carbide tools. Nevertheless, it is crucial to acknowledge that although the micro-machining procedure effectively improved the surface of the WC sample, it did not completely eliminate the prominent burrs and holes that were initially found in raw samples (Figure 2h,i,k,l). The presence of these burrs, despite being less noticeable after undergoing treatment, continued to exist, indicating possible difficulties for applications requiring a flawless surface (Figure 3a).

**Figure 4:**
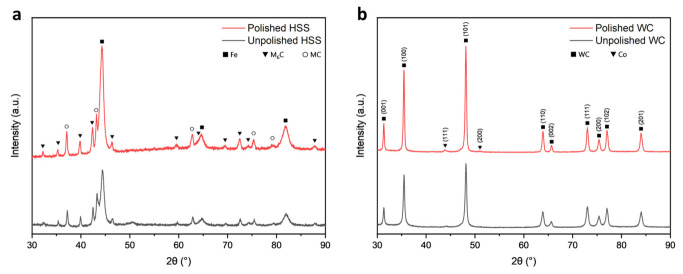
XRD patterns of (**a**) HSS and (**b**) WC sample on unpolished and polished area.

The inner structural modification of hard tool samples was looked into through the use of XRD analysis (Figure 4). The diffractograms obtained from the unpolished HSS samples revealed the presence of distinct basal planes associated with the M2 steel material. These basal planes were identified with three strong peaks that corresponded to α-Fe [38]. Additionally, the diffractograms exhibited supplementary peaks that were attributed to the M_6_C and MC carbide phases, which are often observed in alloyed steels [39]. Interestingly, both unpolished and polished samples exhibited equivalent peak positions. However, the samples that underwent micro-machining displayed narrower and more intense peaks. The observed contrast was found to be less apparent in the HSS samples, but more noticeable in the WC samples. This indicates that the micro-machining procedure likely diminished surface defects and crystalline disorders, resulting in a clearer and more organised underlying crystalline structure. Furthermore, it was observed that the polished sample of WC displayed a greater degree of differentiation in the positions of cobalt peaks when compared to the unpolished one. The aforementioned observation is consistent with the higher cobalt content reported in the EDX investigation (Supplementary Information), hence confirming the consistent nature of the material’s composition. The WC sample exhibited its characteristic patterns as in previous reports [40,41].

**Figure 5:**
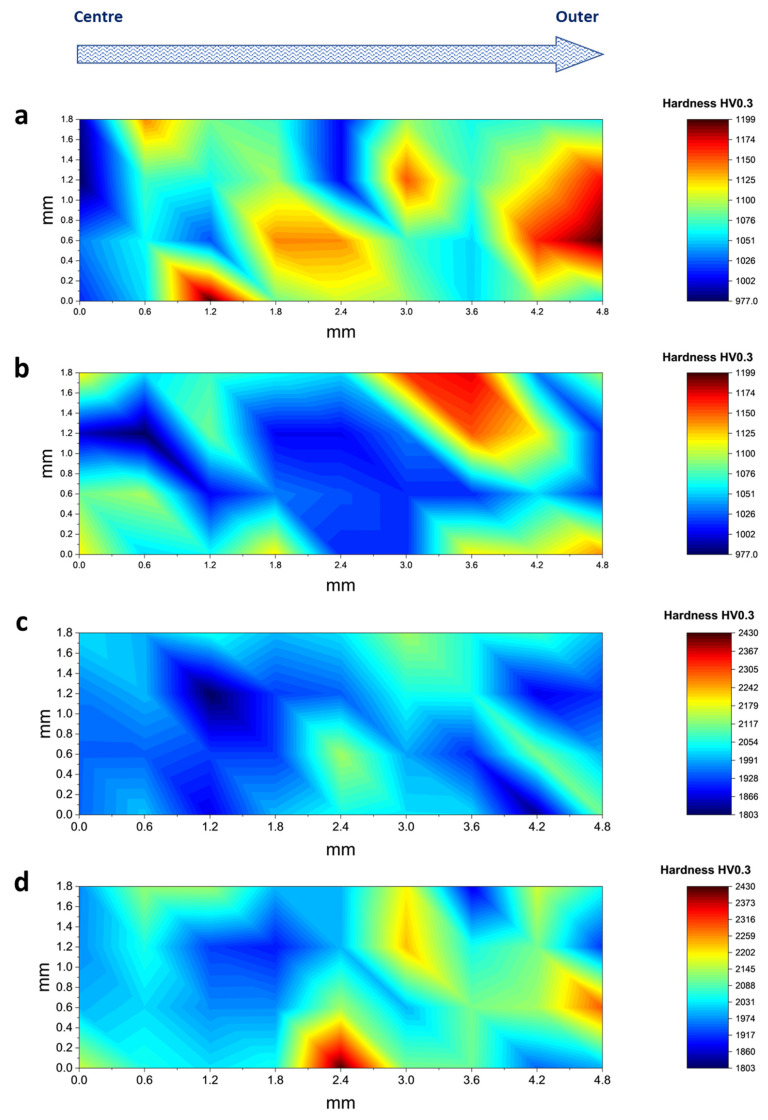
Hardness measurement from centre to periphery part of cross-sectional region on (**a**) unpolished and (**b**) polished HSS sample; and (**c**) unpolished and (**d**) polished WC sample.

The observed variations in elemental composition could impact the mechanical characteristics along the radial gradient of the tool materials. As an example, the high-speed steel sample in its unpolished state displayed an average hardness of 1082.2 ± 54.1 HV. After polishing, the hardness slightly decreased to 1063.4 ± 51.6 HV. This indicates that the fundamental mechanical properties of the HSS samples were mostly unaffected, as supported by the uniform distribution of microhardness from the centre to the periphery regions (Figure 5a,b). In contrast, the unpolished WC sample had an average hardness of 1989.0 ± 79.4 HV. Interestingly, this hardness increased to 2060.4 ± 113.19 HV after the polishing process (Figure 5c,d). The observed increase in hardness can be ascribed to a cryogenic pre-heat treatment procedure, which appears to have improved the hardness in the vicinity of the tool’s tip—the area from which the polished slide was obtained [42]. However, similar to the behaviour observed in HSS, the radial distribution of microhardness in both polished and unpolished WC samples demonstrated a high level of consistency. Therefore, drawing from thorough elemental and mechanical analyses, we hypothesised that the micro-machining process primarily impacts the external surface characteristics, as previously discussed, while causing minor disruptions to the interior structural integrity of the tool.

**Figure 6:**
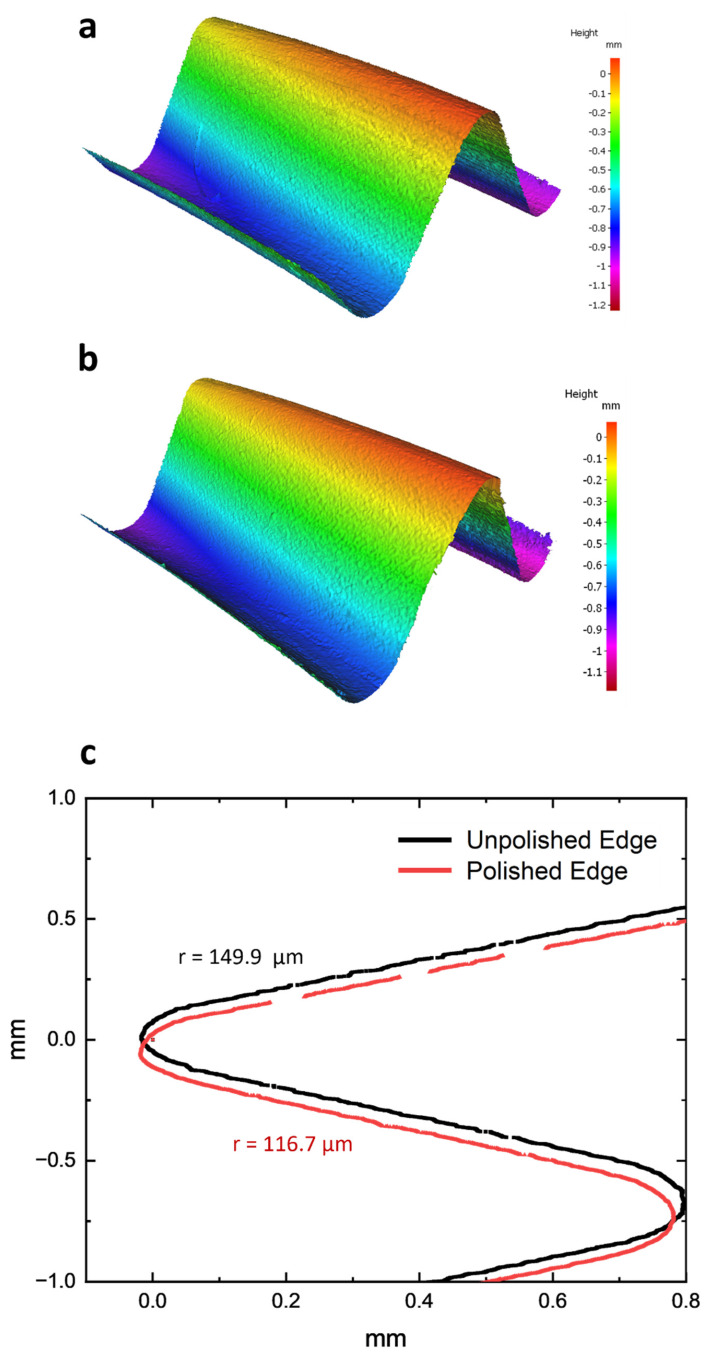
Edge contour 3D scan of (**a**) unpolished and (**b**) polished HSS sample. (**c**) Edge radius measurement with corresponding average radius values of unpolished and polished HSS sample.

**Figure 7:**
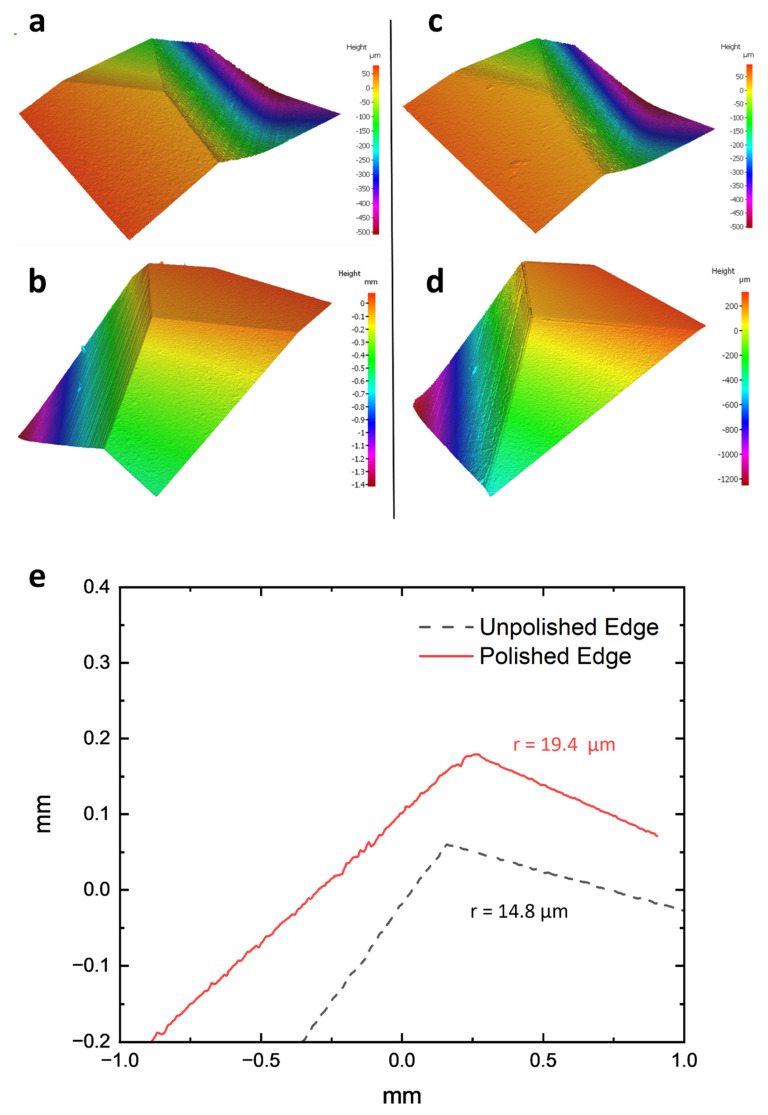
Edge contour 3D scan of (**a**,**b**) unpolished and (**c**,**d**) polished WC sample. (**e**) Edge radius measurement with corresponding average radius values of unpolished and polished WC sample.

**Figure 8:**
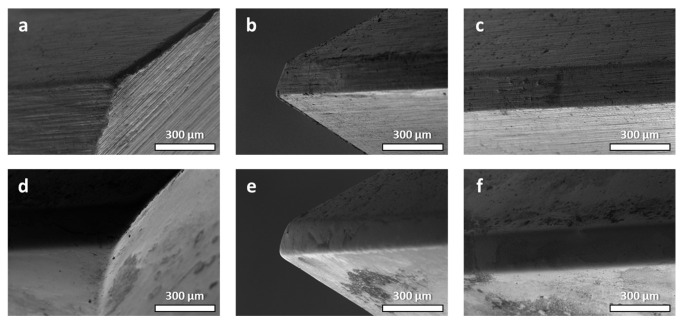
SEM images of primary and secondary cutting edges of HSS tool sample (**a**–**c**) before and (**d**–**f**) after micro-machining process.

**Figure 9:**
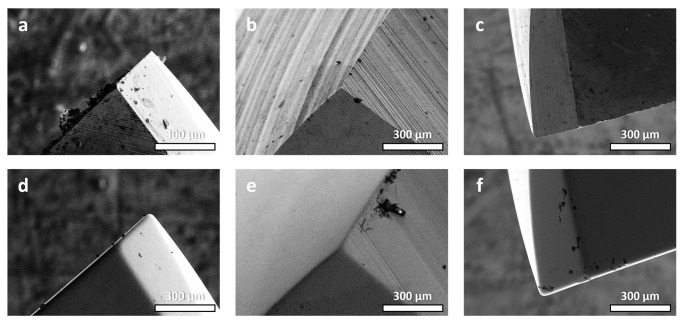
SEM images of primary and secondary cutting edges of WC tool sample (**a**–**c**) before and (**d**–**f**) after micro-machining process.

Aside from the obvious improvement in surface finishing, the micro-machining method also provides considerable edge-honing benefits. To quantify these impacts, 3D metrological techniques were used to inspect the cutting edges of both unpolished and polished tools. The results show a more rounded cutting-edge radius for the HSS tool, with less evidence of micro-chipping. There was a specific increase from 116.7 µm to 149.9 µm (Figure 6). Despite this improvement, the presence of larger burrs was still noticeable. Similarly, the tungsten carbide tool improved edge roundness, with its radius increasing from 14.8 µm to 19.4 µm (Figure 7). Even after machining, the problem of micro-chipping remained. This edge-rounding phenomena could be interpreted as an unintentional result of the micro-machining process’s principal surface finishing goals. Detailed 3D scans revealed the existence of considerable grinding lines and pronounced burrs in both the untreated and treated specimens, a feature that was predicted to disappear after the micro-machining intervention. The observation of alteration effects in edge rounding and surface finish can be enhanced with SEM images. Figure 8 and Figure 9 provide side-by-side comparisons of the primary and secondary cutting edges before and after the micro-machining process. The presented photos depict an impressive reduction in grinding textures observed on samples fabricated from both HSS and WC tool samples. The observed phenomena extended beyond mere enhancements in aesthetics, as it was accompanied with a notable reduction in the occurrence of micro-chipping residues subsequent to the micro-machining processes. The presence of residues, which have the tendency to negatively impact the performance of cutting tools, was reduced to a minimum, suggesting the implementation of an efficient polishing process. The surfaces of both the micro-machined samples exhibit a distinct smoothness, free from any imperfections that may have potentially impacted their functional capabilities.

**Figure 10:**
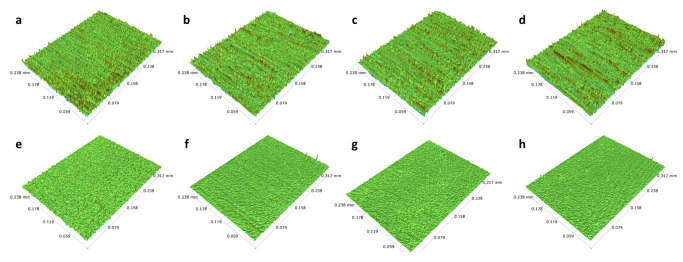
Three-dimensional profilometer scan of (**a**–**d**) before and (**e**–**h**) after micro-machining process of additional HSS tool samples as in Table 1. (**a**,**e**) HSS 2, (**b**,**f**) HSS 3, (**c**,**g**) HSS 4 and (**d**,**h**) HSS 5.

In addition to employing the primary WC and HSS tools, we also submitted a range of alternative tools to diverse conditions for the purpose of micro-machining. The results of these additional examinations are presented in Table 1, with the first two carbide and steel tools representing the above-studied samples. The degree of reduction in roughness may have varied from sample to sample due to the various techniques used, but all samples showed a consistent and noticeable decrease in surface roughness, as can be seen in Figure 10. The finest tool sample was able to attain the surface roughness of about 43.6 nm with relatively low Rz of 1.0 µm.

## 4. Conclusions

The micro-machining procedure performed on samples of high-speed steel and tungsten carbide results in significant alterations to the surface, while simultaneously exerting subtle influences on the internal structure. The HSS tool exhibits notable improvements in surface quality, as evidenced by enhanced clarity and glossiness in the finishes. These improvements are accompanied by substantial reductions in the roughness values, specifically Ra and Rz. On the other hand, the WC tool, despite its dramatically improved smoothness, exhibits a more profound and obscure polished appearance. It is worth mentioning that the utilisation of micro-machining techniques in tungsten carbide has been seen to enhance the leaching of cobalt, leading to the formation of structural voids and a decrease in hardness. Both HSS and WC tools undergo an edge-honing effect, which results in a more rounded cutting edge. This unintentional by-product of surface finishing has the potential to extend the lifespan of the tool. To summarise, micro-machining is a process that enhances the surface characteristics of the cutting tools, resulting in a noticeable decrease in roughness and an overall improvement in the surface quality of the cutting tools. However, it is important to note that the effects of the process are not only limited to the surface alterations, but also to the change in chemical composition that could result in an enhancement or reduction in mechanical properties of the cutting tools. Nevertheless, the presence of continuous grinding lines, burrs, and unwanted edge rounding serves as a reminder of the imperative for precision and meticulousness when utilising micro-machining techniques in the realm of tool making.

## Figures and Tables

**Table 1:** Surface roughness of different micro-machined tool samples.

Sample	Unpolished				Polished			
	R_a_ (nm)	R_q_ (nm)	R_z_ (µm)	R_p_ (µm)	R_a_ (nm)	R_q_ (nm)	R_z_ (µm)	R_p_ (µm)
WC	396.0 ± 13.8	564.2 ± 21.3	8.6 ± 0.6	10.1 ± 1.0	63.7 ± 9.9	85.4 ± 12.9	1.5 ± 0.5	2.1 ± 0.7
HSS	269.0 ± 16.2	376.9 ± 27.3	7.5 ± 1.0	9.9 ± 1.5	74.3 ± 10.3	102.3 ± 17.1	3.5 ± 1.1	4.0 ± 1.2
HSS 2	297.0 ± 21.3	424.8 ± 33.8	7.7 ± 0.7	9.4 ± 1.9	155.3 ± 13.8	224.4 ± 22.6	5.2 ± 1.2	6.8 ± 1.9
HSS 3	283.3 ± 10.1	395.5 ± 14.4	6.1 ± 0.8	7.2 ± 1.2	43.6 ± 4.0	57.7 ± 9.1	1.0 ± 0.8	1.9 ± 1.2
HSS 4	295.2 ± 26.0	425.4 ± 35.9	8.2 ± 0.8	9.9 ± 1.5	52.7 ± 5.1	79.2 ± 10.6	2.4 ± 0.4	3.9 ± 0.5
HSS 5	269.3 ± 16.3	376.9 ± 27.1	6.1 ± 0.7	7.5 ± 1.0	66.2 ± 9.7	92.1 ± 16.0	2.2 ± 0.8	3.3 ± 1.0

## Data Availability

Not applicable.

## Supplementary Materials

The following supporting information can be downloaded at: https://www.mdpi.com/article/10.3390/mi14101970/s1, Figure S1: Elemental analysis by EDX from centre to periphery part of cross-sectional HSS sample on Unpolished and Polished area. The EDX tables at the bottom represent the outer surface of unpolishing and polished area correspondingly; Figure S2: Elemental analysis by EDX from centre to periphery part of cross-sectional WC sample on Unpolished and Polished area. The EDX tables at the bottom represent the outer surface of unpolishing and polished area correspondingly.
