# Supplementary material for: An Investigation into the Surface Integrity of Micro-Machined High-Speed Steel and Tungsten Carbide Cutting Tools
Source: Micromachines (Basel). 2023 Oct 22;14(10):1970. doi: 10.3390/mi14101970 (PMC10608892; doi:10.3390/mi14101970)
Supplement: Supplementary file 1 [file micromachines-14-01970-s001.zip › micromachines-2602336-supplementary.pdf]

Supplementary Information

An investigation into the surface integrity of micro-machined high-speed steel and tungsten carbide cutting tools

Minh Nhat Dang [1*], Surinder Singh [1], John H. Navarro-Devia [2], Hannah J. King [1], Rosalie K. Hocking [1], Scott A. Wade [1], Guy Stephens [2], Angelo Papageorgiou [2], and James Wang [1*]

[1] *The Australian Research Council (ARC) Training Centre in Surface Engineering for Advanced Materials (SEAM), School of Engineering, Swinburne University of Technology, PO Box 218, Hawthorn VIC 3122, Australia.*
[2] *Sutton Tools, 378 Settlement Rd, Thomastown VIC 3074, Australia.*

Email: nhatminh@swin.edu.au; jawang@swin.edu.au

Energy Dispersive X-ray Spectroscopy was utilised to conduct elemental analysis on the cross-sectional specimens and outer surface of high-speed steel and tungsten carbide tools. A noticeable pattern in the distribution of elements was identified, with a clear organisation apparent from the centre to periphery regions in both HSS and WC samples. Quantitative analysis indicated that the cobalt content in the polished area of the HSS sample exhibited a slight decrease, whereas the WC sample exhibited an elevation in cobalt concentration. The persistence of constituent distribution in an apparently random pattern inside the homogeneous matrix of both HSS and WC alloys is a subject of intrigue. It is worth mentioning that a substantial decrease of roughly 13 wt.% was seen in the iron content of the HSS sample after micro-machining (Figure S1).This reduction was accompanied by an increase in the concentrations of vanadium, molybdenum, and mostly tungsten. In contrast, the composition of tungsten in the WC sample exhibited minimal changes after the machining process (Figure S2). These notable shifts in elemental composition raise the possibility of a leaching issue, which could contribute to the minor changes in the hardness of the post-machining samples.

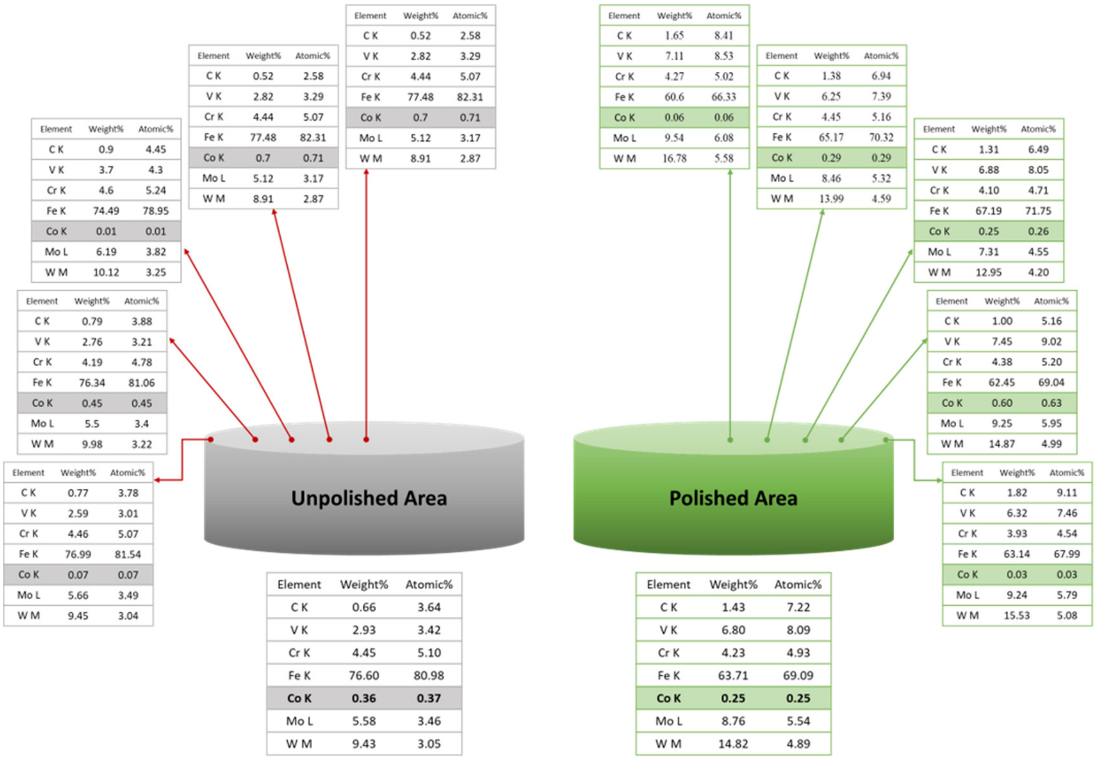

Figure S1. Elemental analysis by EDX from centre to periphery part of cross-sectional HSS sample on Unpolished and Polished area. The EDX tables at the bottom represent the outer surface of unpolishing and polished area correspondingly.

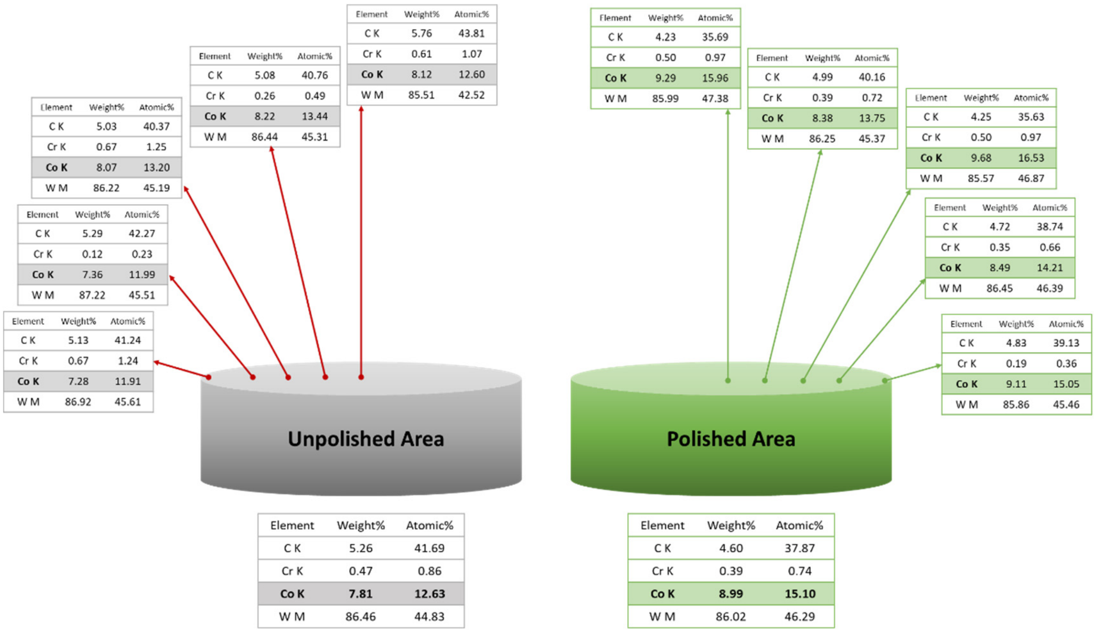

Figure S2. Elemental analysis by EDX from centre to periphery part of cross-sectional WC sample on Unpolished and Polished area. The EDX tables at the bottom represent the outer surface of unpolishing and polished area correspondingly.
